# Determination of key hub genes in Leishmaniasis as potential factors in diagnosis and treatment based on a bioinformatics study

**DOI:** 10.1038/s41598-024-73779-w

**Published:** 2024-09-28

**Authors:** Mohsen Safaei, Arash Goodarzi, Zahra Abpeikar, Ahmad Reza Farmani, Seyed Amin Kouhpayeh, Sohrab Najafipour, Mohammad Hassan Jafari Najaf Abadi

**Affiliations:** 1 Department of Tissue Engineering, School of Advanced Technologies in Medicine, Fasa University of Medical Sciences, Fasa, Iran; 2 Department of Pharmacology, School of Medicine, Fasa University of Medical Sciences, Fasa, Iran; 3 Department of Microbiology, Faculty of Medicine, Fasa University of Medical Sciences, Fasa, Iran; 4 Department of Medical Biotechnology, School of Medicine, Shahid Sadoughi University of Medical Sciences and Health Services, Yazd, Iran; 5 Research Center for Health Technology Assessment and Medical Informatics, School of Public Health, Shahid Sadoughi University of Medical Sciences, Yazd, Iran

**Keywords:** Leishmaniasis, Differentially expressed genes, RRA method, Microarray, GEO database, Drug discovery, Immunology, Microbiology, Molecular biology, Systems biology, Biomarkers, Diseases, Pathogenesis, Mathematics and computing

## Abstract

Leishmaniasis is an infectious disease caused by protozoan parasites from different species of leishmania. The disease is transmitted by female sandflies that carry these parasites. In this study, datasets on leishmaniasis published in the GEO database were analyzed and summarized. The analysis in all three datasets (GSE43880, GSE55664, and GSE63931) used in this study has been performed on the skin wounds of patients infected with a clinical form of leishmania (*Leishmania braziliensis*), and biopsies have been taken from them. To identify differentially expressed genes (DEGs) between leishmaniasis patients and controls, the robust rank aggregation (RRA) procedure was applied. We performed gene functional annotation and protein-protein interaction (PPI) network analysis to demonstrate the putative functionalities of the DEGs. The study utilized Molecular Complex Detection (MCODE), Gene Ontology (GO), and Kyoto Encyclopedia of Genes and Genomes (KEGG) to detect molecular complexes within the protein-protein interaction (PPI) network and conduct analyses on the identified functional modules. The CytoHubba plugin’s results were paired with RRA analysis to determine the hub genes. Finally, the interaction between miRNAs and hub genes was predicted. Based on the RRA integrated analysis, 407 DEGs were identified (263 up-regulated genes and 144 down-regulated genes). The top three modules were listed after creating the PPI network via the MCODE plug. Seven hub genes were found using the CytoHubba app and RRA: CXCL10, GBP1, GNLY, GZMA, GZMB, NKG7, and UBD. According to our enrichment analysis, these functional modules were primarily associated with immune pathways, cytokine activity/signaling pathways, and inflammation pathways. However, a UBD hub gene is interestingly involved in the ubiquitination pathways of pathogenesis. The mirNet database predicted the hub gene’s interaction with miRNAs, and results revealed that several miRNAs, including mir-146a-5p, crucial in fighting pathogenesis. The key hub genes discovered in this work may be considered as potential biomarkers in diagnosis, development of agonists/antagonist, novel vaccine design, and will greatly contribute to clinical studies in the future.

**Supplementary Information:**

The online version contains supplementary material available at 10.1038/s41598-024-73779-w.

## Introduction

According to the findings of the World Health Organization (WHO), leishmaniasis is an important and neglected tropical disease that mainly affects poor populations in developing and less developed countries^1,2^. This disease is a zoonosis caused by protozoa of the genus Leishmania and transmitted to humans by the bite of an infected mosquito. The main reservoirs of the disease are dogs and cats. The clinical manifestations of the disease vary, depending on the type of leishmania infecting, geographic location, and immune status^3,4^. Based on this, the disease has three main clinical forms, including mucocutaneous leishmaniasis, visceral leishmaniasis (VL), and cutaneous leishmaniasis (CL)^5^. The most common species in Latin America that causes American tegumentary leishmaniasis (ATL) is *Leishmania* (Viannia) *braziliensis*. The clinical symptoms of *L. braziliensis* infections vary from self-healing skin lesions to chronic wounds and mucosal complications. The main target cells that parasites employ for intracellular growth and survival are the macrophages^6^. It is reported that the number of deaths caused by leishmaniasis in the world is between 20,000 and 40,000 cases per year^7,8^.

Currently, it is not cost-effective to examine the expression changes of all genes at the laboratory level, and it is better to perform the initial screening on the data of functional genome studies. Therefore, bioinformatics tools to select genes with distinct expression in disease samples can be helpful^9,10^. Recent advancements in sequencing technology, such as next-generation sequencing (NGS) and high-throughput sequencing methods, have opened up innovative avenues for diagnosing and managing infections. Examining transcriptomic data obtained from infected cells plays a crucial role in pinpointing the root of the disease and selecting the most suitable treatment approach^11^. Efficient techniques based on bioinformatics, such as microarray or machine learning, can reduce costs and infrastructure in the project and also provide ease of analysis. Meanwhile, in some cases, the use of bioinformatics approaches can be an in-silico alternative to animal models for evaluating drug toxicity, thus reducing expensive and invasive animal testing during clinical trials for drugs that are likely to fail safety trials^12,13^.

There are many methods to check the expression of genes, and one of the most important of these methods is the microarray. Microarray technology can be a powerful tool for discovering genes, investigating their structure and function, and discovering pathways involved in disease, which can help diagnose and treat. This study investigates the network of interactions between proteins. Also, to better understand the biological significance of the hub genes in leishmaniasis, the interaction between miRNAs and hub genes was predicted. We also investigated a drug interaction with hub genes via different databases. The investigation of the networks will make us aware of the mechanism of the disease’s action, and clarify the molecular basis and biological details related to the disease and ultimately lead to prevention, diagnosis, treatment, and drug design^14,15^.

## Results

### Characteristics of the selected microarrays

**Fig. 1:**
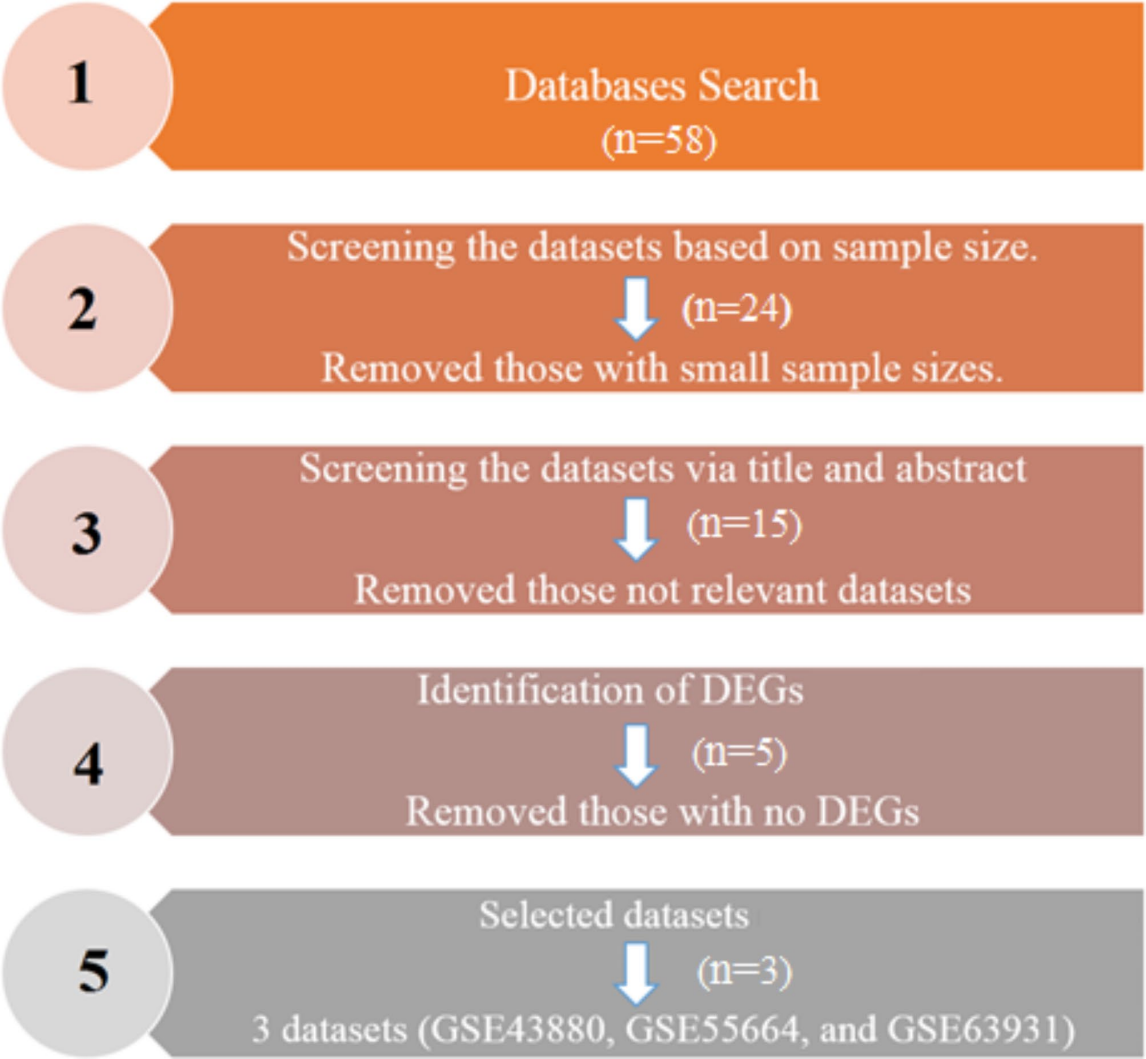
Flowchart for dataset retrieval and selection.

Following the outlined criteria, three datasets were ultimately incorporated into the conclusive analysis: GSE43880^16^, GSE55664^17^, and GSE63931^18^. The GSE43880dataset contains 12 skin biopsy samples, including 2 normal samples from North America and 10 from *L. brazilensis* infected patients at Corte de Pedra Health Post, Bahia, Brazil^16^. Regarding GSE55664 dataset, 35 skin biopsies have been evaluated, which include 10 normal skin biopsies (8 from a non-endemic area in Brazil and 2 from North America) and 25 skin lesion biopsies (17 late cutaneous lesions and 8 early cutaneous lesions) collected from *L. brazilensis*-infected patients who presented at the Corte de Pedra Health Post (Bahia, Brazil)^17^. Based on GSE63931 dataset, the study included 8 skin ulcers from patients infected with *L. braziliensis*. The patients chosen for the gene expression investigation had recently contracted *L. braziliensis* infection but had not yet been treated. The 8 control samples are skin biopsies from healthy donors who are not infected^18^. Figure 1 illustrates the process of dataset selection, encompassing inclusion and exclusion criteria. Out of these three datasets, the experimental group surrounded 43 instances of leishmaniasis, and the standard control group contained 20 cases. The attributes and specifics of these integrated microarray datasets can be found in Table 1.

**Table 1:** Characteristics of the included microarray datasets.

GSE ID	Participants (control/leishmania)	Analysis type	Platform	Year	Tissues	Clinical form of leishmania
GSE63931	8/8	Array	GPL17077	2015	Skin	*L. braziliensis*
GSE55664	10/25	Array	GPL10558	2014	Skin	*L. braziliensis*
GSE43880	2/10	Array	GPL10558	2013	Skin	*L. braziliensis*

### DEGs identification in leishmaniasis

**Fig. 2:**
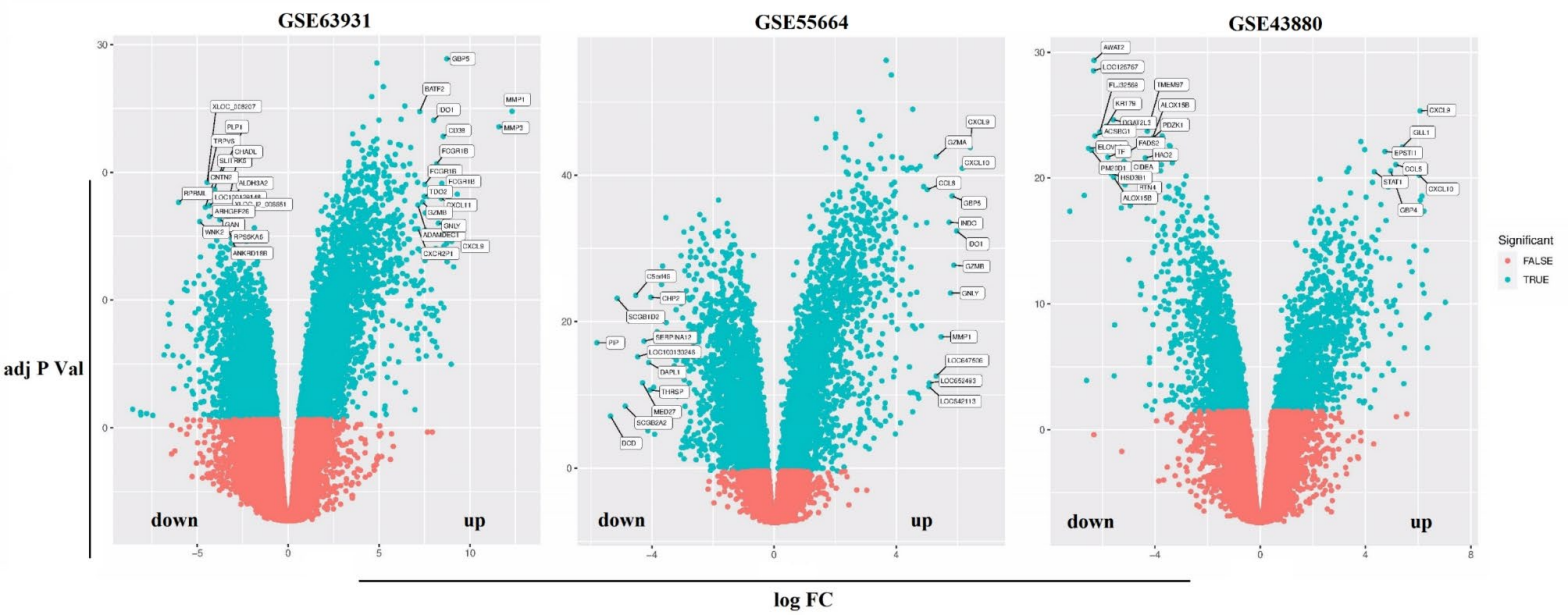
Volcano maps of the three datasets. The location of up-regulated and down-regulated genes is indicated in the Figure. Red points indicate genes with no significant difference. The Figure was obtained by R software.

Initially, DEGs in leishmaniasis were determined. Initially, the datasets underwent standardization to address batch differences, as depicted in Supplementary Fig. 1, confirming data consistency for analysis. Subsequently, DEGs were pinpointed within each dataset by the Limma package of R software, with corresponding volcano maps displayed in Fig. 2.

### Analysis of RRA integrated

**Fig. 3:**
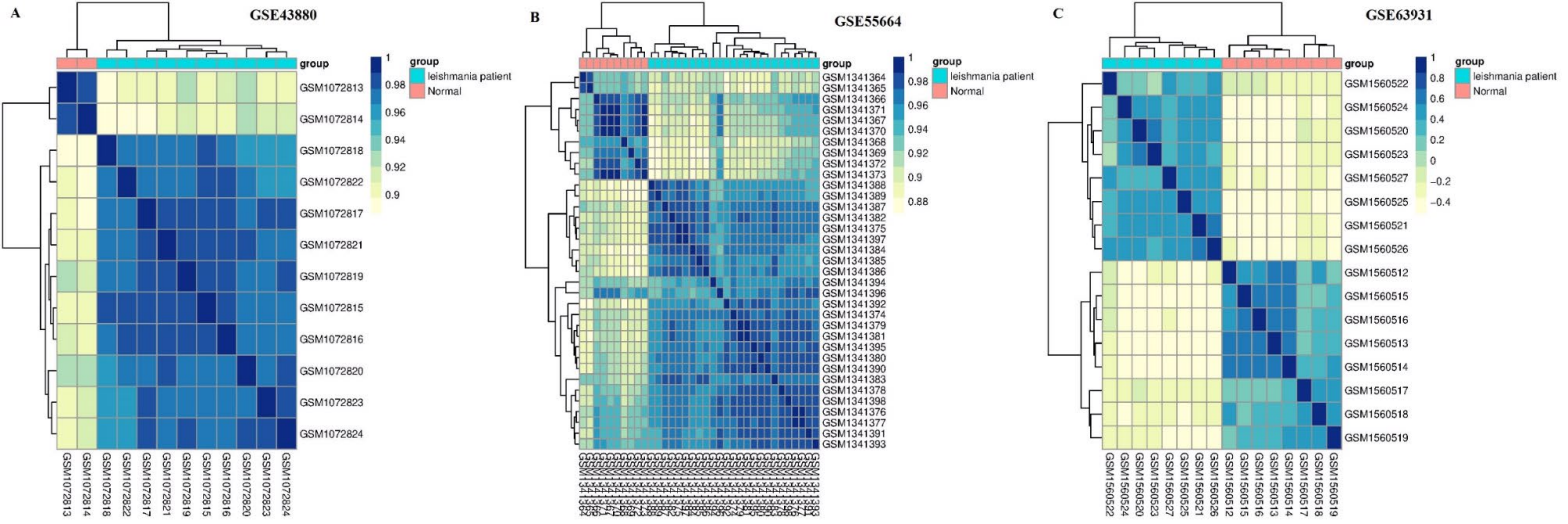
The heatmap diagram. DEGs Clustering between the control/normal (Red color) and leishmaniasis groups (Turquoise blue color). The Figure was obtained by R software.

**Fig. 4:**
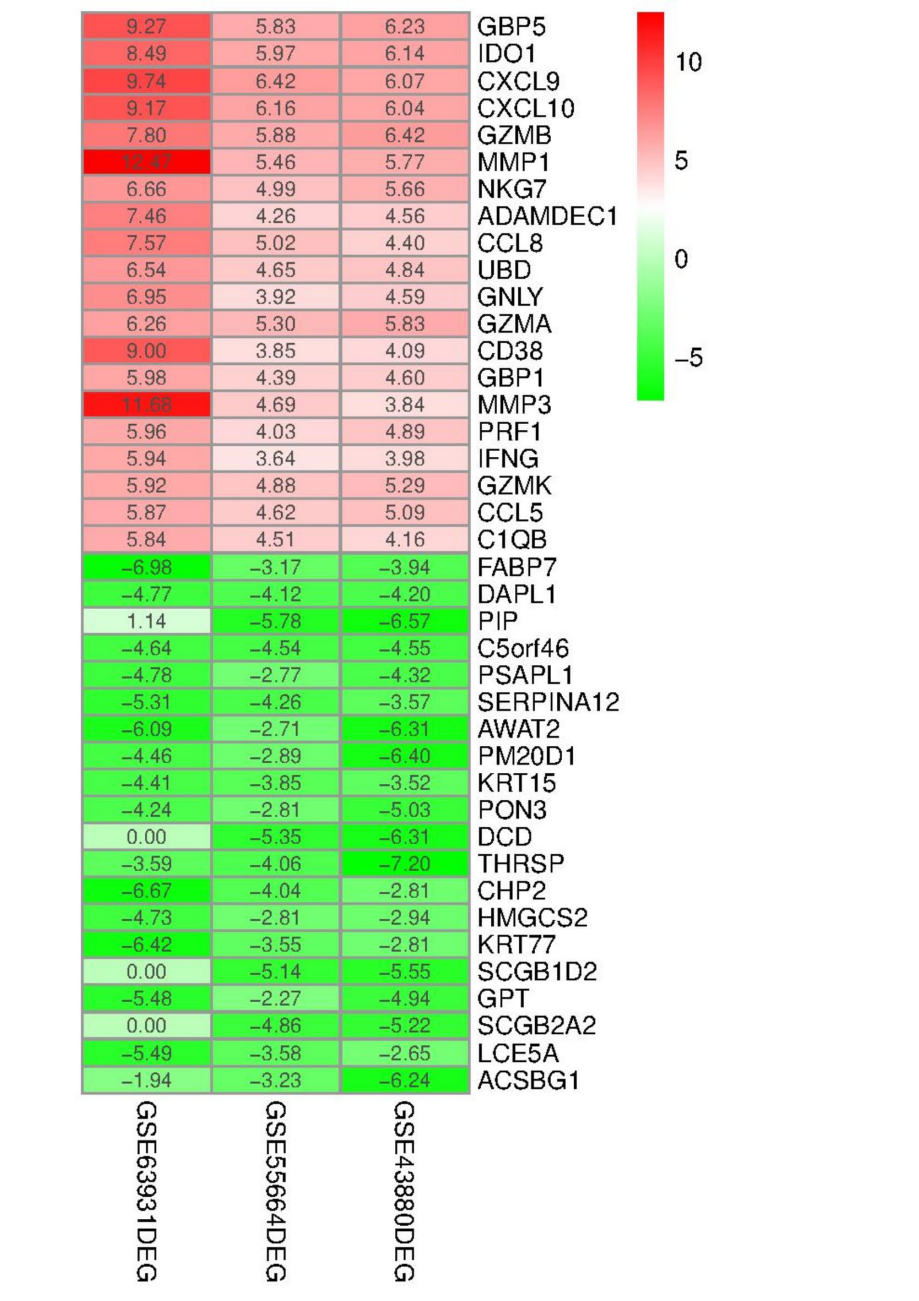
Heatmap exhibiting the top 20 DEGs (up-regulated or down-regulated) found in the RRA analysis. Red indicates a relatively high expression of genes in Leishmania patients, and green indicates a relatively low expression of genes in Leishmania patients. The values in the heatmap indicate the logarithmic fold change in each dataset, as estimated by R software. The Figure was obtained by R software.

The heatmap diagram related to the three datasets included in the study based on clustering between the control and leishmaniasis groups is shown in Fig. 3. In this Figure, the clustering of DEGs between the two mentioned groups is well-defined. A total of 407 DEGs (263 up-regulated and 144 down-regulated) were detected through RRA analysis. Figure 4 displays a heatmap of the top 20 DEGs, whether up-regulated or down-regulated. Comprehensive results from the RRA analysis can be found in Supplementary Table 1.

Among the ten most significant genes with abnormal expression in leishmaniasis, five genes were up-regulated [GBP5 (*P* = 3.79E−08), IDO1 (*P* = 2.21E−07), CXCL9 (*P* = 3.03E−07), CXCL10 (*P* = 4.03E−07), GZMB (*P* = 5.24E-07)], and five genes were down-regulated [FABP7 (*P* = 1.05E–05), DAPL1 (*P* = 1.25E−05), PIP (*P* = 1.62E−05), C5orf46 (*P* = 2.61E–05), PSAPL1 (*P* = 3.55E−05)].

### Functional annotation

**Fig. 5:**
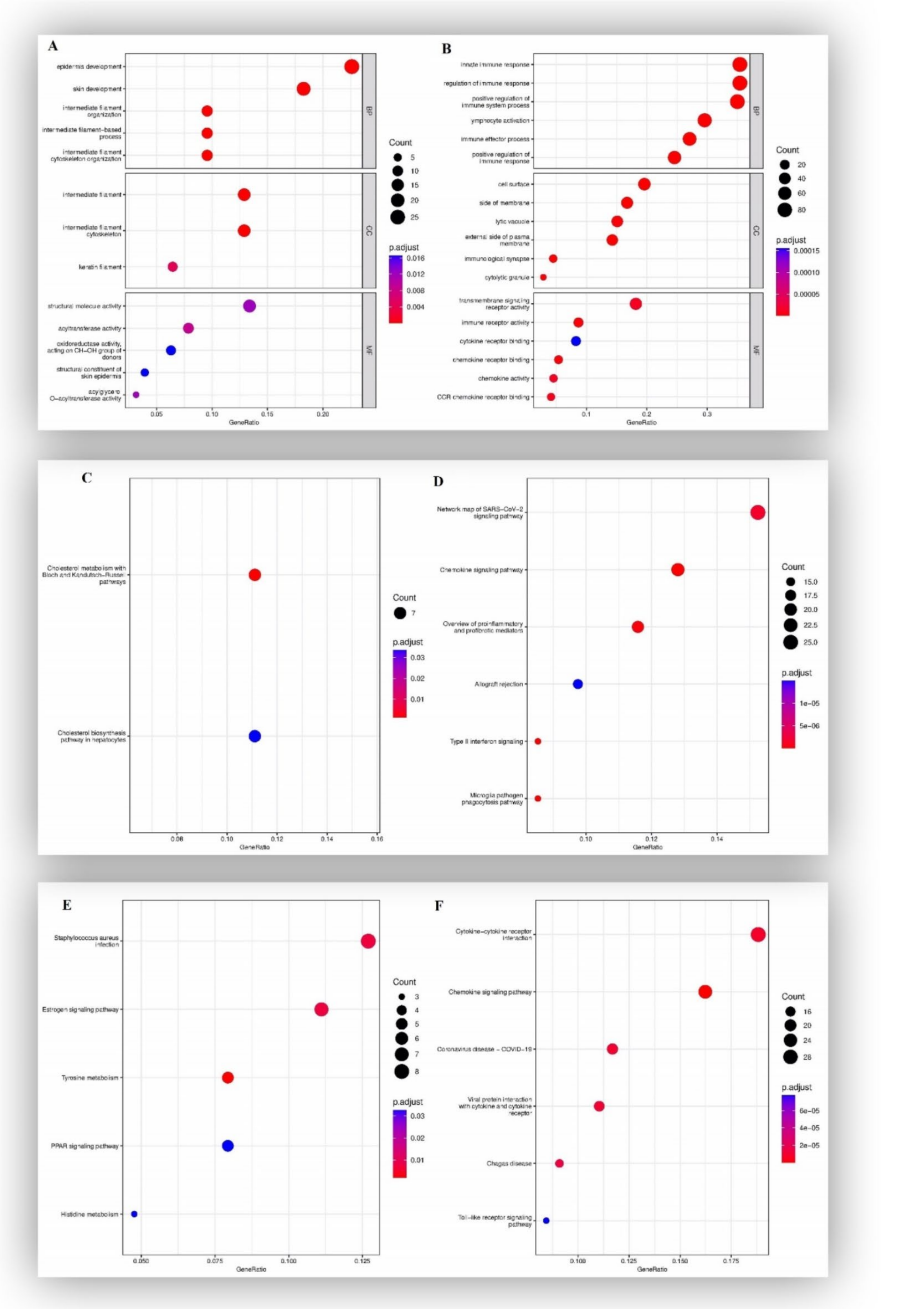
GO analysis of DEGs: (A) Functional enrichment analysis of down-regulated genes. (B) Functional enrichment analysis of up-regulated genes. WIKIpath enrichment analysis of DEGs: (C) Functional enrichment analysis of downregulated genes. (D) Functional enrichment analysis of up-regulated genes. KEGG pathway enrichment analysis for DEGs^19,20^: (E) The functional enrichment analysis of down-regulated genes. (F) Functional enrichment analysis of up-regulated genes. The Figures were obtained by R software.

For analysis, 263 up-regulated and 144 down-regulated DEGs were uploaded, including GO (covering cellular components, molecular function, and biological process), WIKIpath enrichment, and KEGG analysis. The findings revealed that up-regulated DEGs were notably associated with terms like innate immune response, immune response regulation, lymphocyte activation, and receptor activity, with the top three enriched terms determined by their respective P-values (as shown in Fig. 5B). In contrast, down-regulated DEGs were predominantly linked to epidermis development, skin development, and filament-related processes based on their P-values (Fig. 5A).

According to the WIKIpath enrichment analysis, the up-regulated DEGs were primarily involved in the network maps related to the SARS–CoV–2 signaling pathway, the chemokine signaling pathway, and an overview of proinflammatory and profibrotic mediators. In contrast, the down-regulated genes were mainly associated with pathways related to cholesterol metabolism, including the Bloch and Kandutsch–Russell pathways and the cholesterol biosynthesis pathway in hepatocytes (as illustrated in Fig. 5C and D).

As indicated by KEGG pathway enrichment, the up-regulated DEGs were predominantly engaged in pathways such as cytokine–cytokine receptor interaction, the chemokine signaling pathway, and those related to coronavirus disease (COVID-19) and Chagas disease. On the other hand, the down-regulated genes were primarily linked to pathways concerning *staphylococcus aureus* infection, the estrogen signaling pathway, and tyrosine metabolism, as depicted in Fig. 5E and F.

### Analysis of PPI networks and hub genes identification

**Fig. 6:**
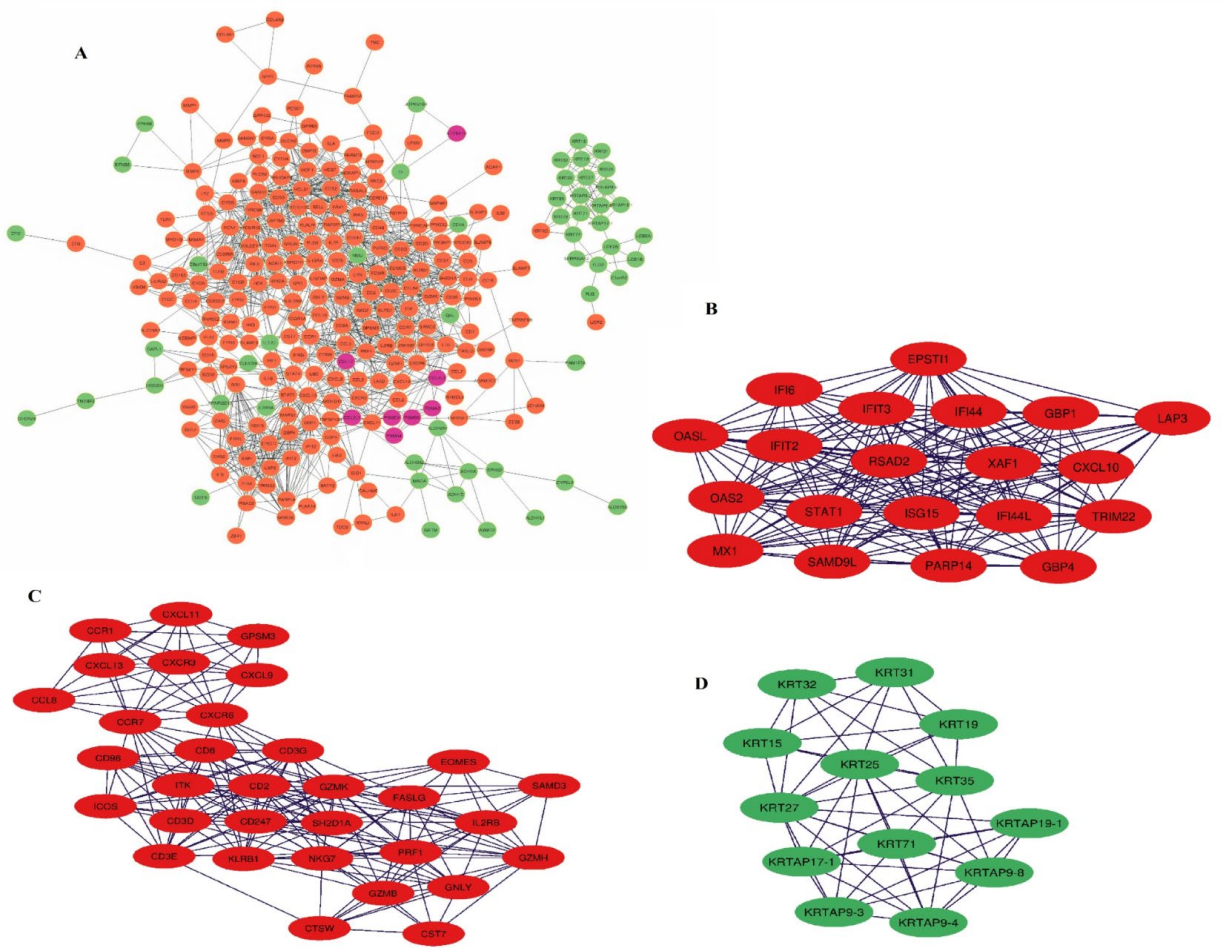
Representation and module determination for the PPI network. (A) Cytoscape software was used to map 407 DEGs. The MCODE plug-in identified three modules of the PPI networks. (B) Module 1 comprised IFI44, ISG15, IFIT2, IFI44L, PARP14, SAMD9L, MX1, IFI6, OAS2, IFIT3, RSAD2, XAF1, STAT1, EPSTI1, OASL, GBP1, GBP4, LAP3, CXCL10 with the seed gene TRIM22; (C) module 2 contained CD3D, GZMB, CXCR6, ITK, GZMH, CD6, CCL8, IL2RB, CD2, SH2D1A, FASLG, NKG7, CTSW, CXCL11, CXCL9, ICOS, CST7, SAMD3, EOMES, CD96, CD247, CXCR3, GPSM3, CD3E, GNLY, CCR1, GZMK, CXCL13, PRF1,CCR7,CD3G with the seed gene KLRB1; (D) module 3 consisted of KRTAP17-1, KRT32, KRT15, KRT35, KRT71, KRT19, KRTAP9-8, KRT25, KRT27, KRTAP9-4, KRT31, KRTAP19-1 with the seed gene KRTAP9-3. The red points indicate up-regulated genes, while the green points indicate down-regulated genes.

Using the String Database website, a visual network of 269 nodes and 1384 edges of DEGs identified by RRA analysis was constructed. After that, the network was uploaded into Cytoscape for further genetic analysis (Fig. 6A). MCODE determined the top three modules with the strongest scores. (Fig. 6B–D). Module 1 comprised IFI44, ISG15, IFIT2, IFI44L, PARP14, SAMD9L, MX1, IFI6, OAS2, IFIT3, RSAD2, XAF1, STAT1, EPSTI1, OASL, GBP1, GBP4, LAP3, CXCL10 with the seed gene TRIM22; module 2 contained CD3D, GZMB, CXCR6, ITK, GZMH, CD6, CCL8, IL2RB, CD2, SH2D1A, FASLG, NKG7, CTSW, CXCL11, CXCL9, ICOS, CST7, SAMD3, EOMES, CD96, CD247, CXCR3, GPSM3, CD3E, GNLY, CCR1,GZMK, CXCL13,PRF1,CCR7,CD3G with the seed gene KLRB1; and module 3 consisted of KRTAP17-1, KRT32, KRT15, KRT35, KRT71, KRT19, KRTAP9-8, KRT25, KRT27, KRTAP9-4, KRT31, KRTAP19-1 with the seed gene KRTAP9-3. Supplementary Table 2 shows the scores for each module.

**Fig. 7:**
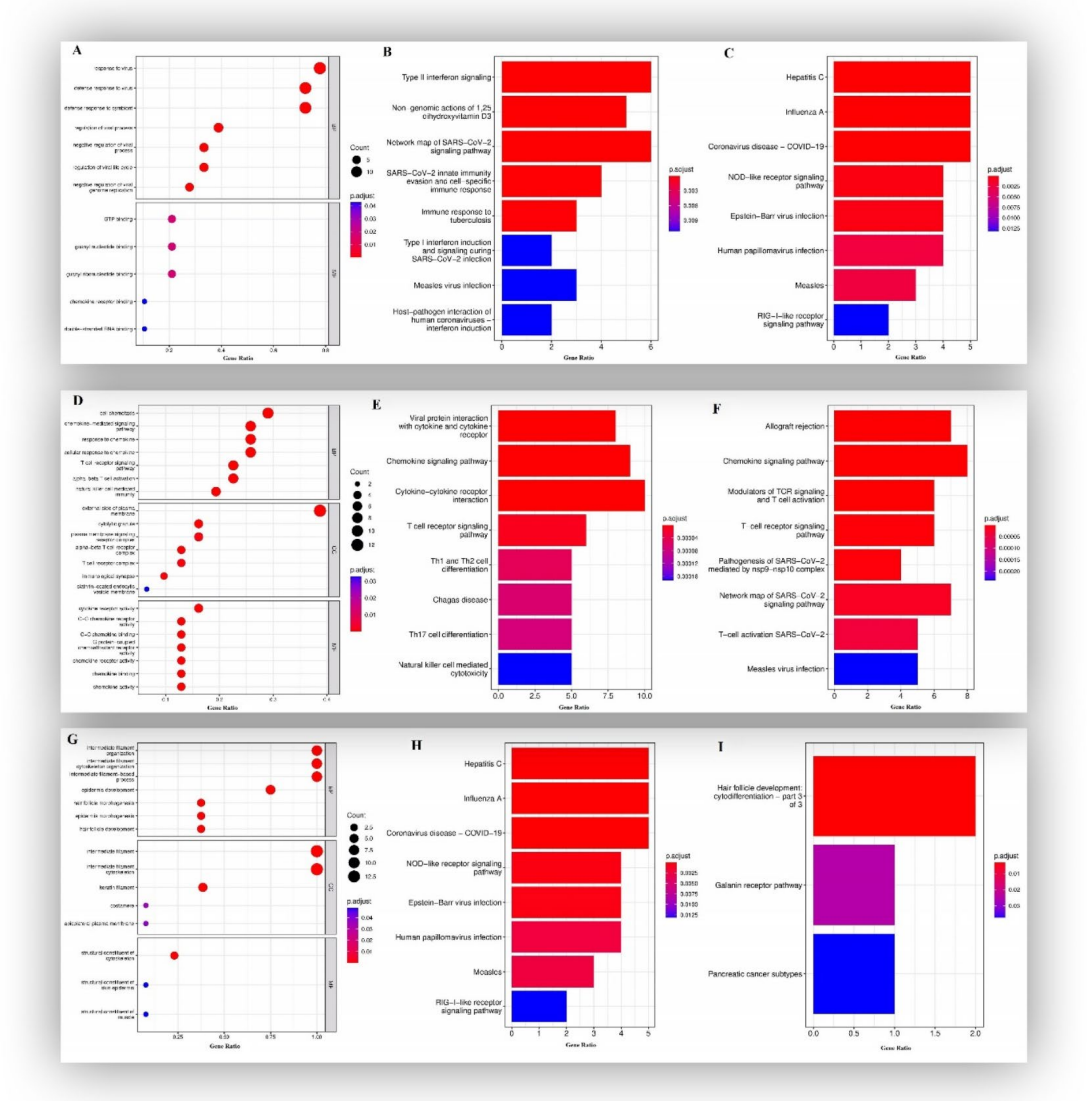
Functional enrichment analysis for the genes in module 1: (A) GO analysis for DEGs. (B) The KEGG analysis for DEGs. (C) The WIKIpath analysis for DEGs. Functional enrichment analysis for the genes in module 2: (D) GO analysis for DEGs. (E) The KEGG analysis for DEGs. (F) The WIKIpath analysis for DEGs. Functional enrichment analysis for the genes in module 3: (G) GO analysis for DEGs. (H) The KEGG analysis for DEGs. (I) The WIKIpath analysis for DEGs^19,20^. The Figures were obtained by R software.

GO enrichment analysis and KEGG analysis of module 1 exhibited that the genes were mainly associated with response to the virus and regulation of viral processes, including (Hepatitis C, Influenza A, and COVID–19) and cytokine activity (Fig. 7A and B), and WIKIpath enrichment analysis revealed that these genes were mainly involved in type II interferon signaling, network map of SARS-CoV-2 signaling pathway and immune response to tuberculosis (Fig. 7C).

GO enrichment and KEGG analysis of module 2 revealed that the DEGs were mainly related to the cell chemotaxis, chemokine-mediated signaling pathway, Cytokine–receptor interaction, and T cell receptor signaling pathway (Fig. 7D and E). The WIKIpath analysis represented that these genes were mainly related to the chemokine signaling pathway, allograft rejection, and modulators of TCR signaling and T cell activation (Fig. 7F).

According to the GO enrichment analysis of module 3, the DEGs were principally associated with the development of the epidermis, the structure of the intermediate filament cytoskeleton, the intermediate filament organization, and the epidermis development (Fig. 7G), and based on KEGG analysis, these genes were primarily implicated in the staphylococcus aureus infection and estrogen signaling pathway (Fig. 7H). Also, WIKIpath analysis displayed that these genes were mainly included in hair follicle development: cytodifferentiation and galanin receptor pathway (Fig. 7I).

### Hub genes determination

CytoHubba was employed to determine critical genes within the protein-protein interaction network, which were subsequently arranged based on their degree scores. By incorporating the findings from the RRA analysis, seven central genes were determined: CXCL10, GBP1, GNLY, GZMA, GZMB, NKG7, and UBD (Table 2). Supplementary Table 3 contains a complete list of all results.

**Table 2:** Key gene hubs selected based on degree, betweenness centrality, and closeness centrality parameters by Cytoscape software.

Gene_name	Degree	Closeness	Betweenness	Clustering Coefficient
GZMA	35	116.45	2388.32	0.36
CXCL10	32	113.28	3712.74	0.34
NKG7	27	111.94	1225.25	0.39
UBD	15	103.35	3061.30	0.20
GZMB	19	99.55	695.21	0.57
GNLY	16	97.25	43.21	0.77
GBP1	22	95.53	878.73	0.55

### Interaction between miRNAs and hub genes

**Fig. 8:**
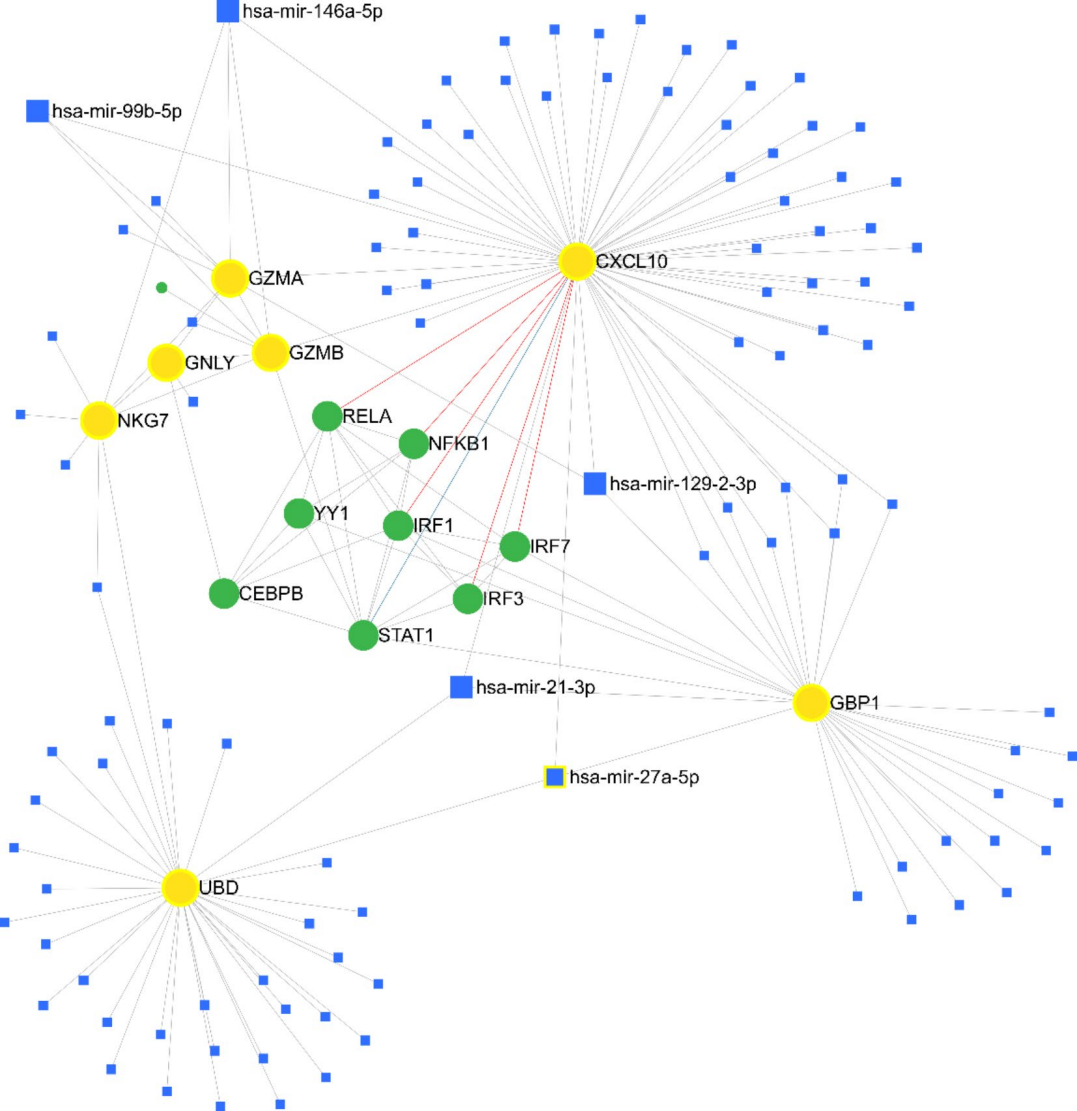
The interaction of miRNAs and selected Hub genes using the the miRNet database.

To better understand the biological significance of these Hub genes in leishmaniasis, the miRNet database suggested possible miRNAs for these genes. There were five miRNAs for these Hub genes (has-mir-146a-5p, has-mir-99b-5p, has-mir-129-2-3p, has-mir-27a-5p, and has-mir-21-3p). For better visualization, the interaction between miRNAs and Hub genes is displayed in a miRNA-Hub gene network in Fig. 8.

### Drug-hub genes interaction

Using the databases mentioned in the materials and methods section, drugs corresponding to the hub genes of the study were predicted. These drugs mostly have inhibitory activity (Table 3).

**Table 3:** Interaction of drugs corresponding to hub genes using databases.

Target	Drug	DrugBank Accession Number	Source of drug-target interaction	Drug Groups	Articles that have reported these drugs in leishmaniasis studies
CXCL10	Clove oil	DB11338	DrugBank	Approved	^21,22^
	Ritonavir	DB00503	DGIdb	Approved	^23,24^
	Zidovudine	DB00495	DGIdb	Approved	^25–27^
GZMB	Cyclosporine	DB00091	DGIdb	Approved	^28–30^
GZMA	Hexachlorophene	DB00756	DGIdb	Approved	^31,32^
CXCL10, GBP1, GNLY, GZMA, GZMB, NKG7	JQ1 compound	DB17021	CTDbase	Investigational	^23^

### Evaluation of hub genes in the early and late phases of the disease

The analysis results of the GSE55664 dataset showed that key hub genes exist in both the early and late stages of the disease and are among the common genes of these stages. Although their fold change rate is higher in the initial phase, they showed some decrease in expression in the delayed phase. (Supplementary Fig. 2; Supplementary Table 4).

**Fig. 9:**
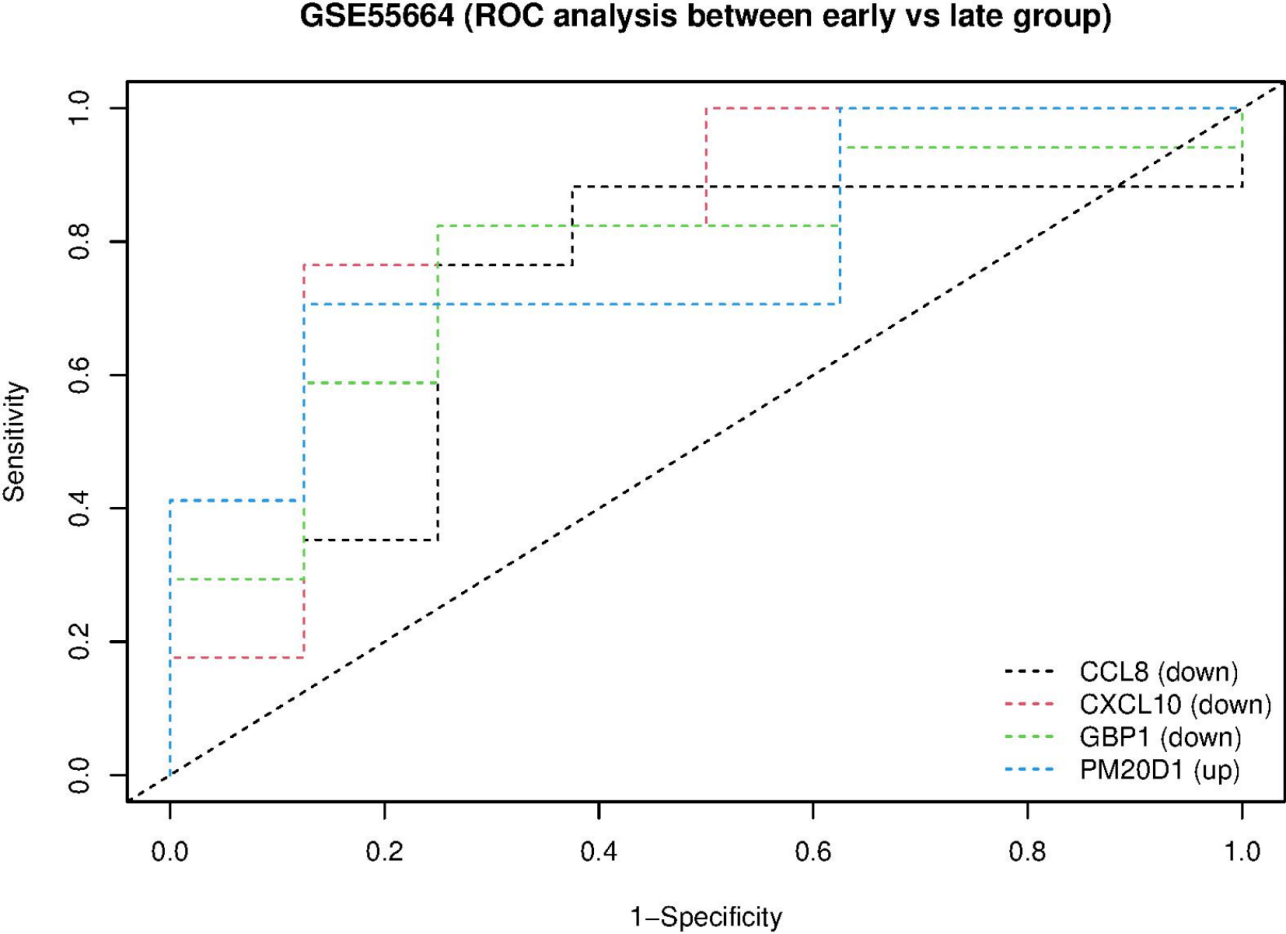
ROC curve analysis of selected genes obtained from sharing three sets of genes (up-regulated DEGs, down-regulated DEGs, and hub genes) in the early vs. late phase related to the GSE55664 dataset. 2 genes out of 4 obtained genes are members of hub genes and are significant at the *P* < 0.05 (CXCL10 and GBP1).

In the GSE55664 dataset, we considered three sets of genes, i.e., up-regulated DEGs, down-regulated DEGs, and hub genes, and these genes were subjected to ROC analysis in two phases, early and late. The results showed that 4 genes shared in this set were significant (*P* < 0.05), and two of these genes are among our hub genes (CXCL10 and GBP1) (Supplementary Fig. 3; Fig. 9).

## Discussion

Various Leishmania species cause cutaneous leishmaniasis, including *L. braziliensis* in the New World and *L. major* in the Old World. *L. braziliensis* can lead to symptoms ranging from mild lesions to severe mucosal damage. Despite its significance, research on the immune mechanisms is limited, and drug resistance complicates treatment^33,34^. Developing new drugs requires a deeper understanding of the disease. Investigating gene pathways and protein networks with the help of bioinformatics and system biology can help discover new drugs for leishmaniasis against challenges such as drug resistance^35,36^. This work integrated various published datasets for bioinformatics analysis.

An analysis of the PPI network was conducted for all DEGs. Multiple functional gene modules were identified using MCODE, an algorithm that automatically predicts protein complexes from qualitative protein-protein interaction data^37^. The hub genes identified in the current study were involved in some pathways, such as responses to viral infections, including COVID-19 (Figs. 5 and 7). Interestingly, these biomarkers were also discovered in separate experimental investigations focused on COVID-19 patients. These biomarkers appear to play a crucial role in the inflammatory pathways associated with the COVID-19 virus. Furthermore, some of the identified hub genes have been proposed as potential diagnostic tools and prognostic indicators for assessing the stage of COVID-19 disease progression^38–43^.

To evaluate gene connectivity and node importance in a biological network, CytoHubba was used to screen DEGs using different algorithms^44^. By incorporating the findings from the RRA analysis, seven central genes were determined: C-X-C motif chemokine ligand 10 (CXCL10), Guanylate-binding protein 1 (GBP1), Granulysin (GNLY), granzyme A (GZMA), granzyme B (GZMB), Natural killer cell granule protein 7 (NKG7), and Ubiquitin D (UBD). Studies have shown that *L. major* decreases extracellular CXCL10 protein levels, suggesting a potential method for evading the adaptive immune response. Additionally, adding CXCL10 to macrophages infected with *L. amazonensis* reduces parasites and lesion size in mice, increasing the production of IFN-g, IL-12, and nitric oxide^45,46^.

The three hub genes (GZMA, GZMB, and GNLY) obtained in this study are classified as cytotoxic proteins. Several investigations have found that cytotoxicity is one of the key mechanisms driving disease caused by *L. braziliensis* infection^47–49^. Campos et al.^50^ found that genes related to cytolysis, such as GZMB, GZMA, and GNLY, were overexpressed in CL lesions compared to normal skin. Studies have shown that localized skin inflammation in cutaneous leishmaniasis leads to a chronic systemic IFN-γ signature, with increased expression of cytotoxic markers GZMB, Pore-forming protein 1(PRF1), and GNLY. IFN-γ activates phagocytic cells like monocytes and macrophages to control parasite replication, while cytotoxicity can heighten inflammation and worsen the disease. Even in localized infections, these gene expression changes indicate both protective and pathogenic roles^51–53^. It has been reported that granule exocytosis of GNLY and GZMB is a potential key mechanism in vaccine-induced immunity in cattle against nematode *Ostertagia ostertagi*^54^. Considering the expression of cytotoxic biomarkers, including granzymes A /B and chemokine CXCL10 in CL, it is possible to use the approach of designing agonists to make potential vaccines, as well as the strategy of considering antagonists to design drugs to modulate immune responses in leishmaniasis. A critical pathway for designing agonists based on the identified hub genes, particularly cytotoxic proteins, is the Toll-like receptor (TLR) pathway. TLRs are pattern recognition receptors linking innate and adaptive immune systems^55^. TLR2 is expressed by antigen-presenting cells (APCs) of the innate immune system, including macrophages, dendritic cells (DCs), etc. It is crucial for detecting parasites and triggering immune responses during Leishmania infection. TLRs also promote cytotoxic T lymphocytes (CTLs), leading to increased levels of cytotoxic proteins and granzymes A/B^56,57^. TLR2/TLR4 antagonists or agonists may offer therapeutic benefits in eliciting an appropriate immune response during leishmaniasis, including *L. braziliensis*; Additionally, TLR agonists can serve as adjuvants in anti-leishmania vaccines^58^. We also performed a drug interaction with hub genes in the present study using different databases. The results showed that most drugs have an inhibitory effect (Table 3). Also, various studies that have reported about the drugs found in these databases for leishmaniasis are referred to in Table 3.

Some identified hub genes play a significant role in the inflammatory process, and their increased expression levels suggest potential use for early disease diagnosis. However, there is a lack of studies focusing on the early and late phases of leishmaniasis, with limited datasets available for comparison. More research is needed based on in vitro and in vivo models. The analysis of the GSE55664 dataset in this study indicates that the key hub genes are present in both the early and late stages of the disease and are common to these stages. Although their expression profile is similar, the fold change rate is higher in the initial phase and decreases in the delayed stage (Supplementary Fig. 2; Supplementary Table 4). The dynamic balance and changes in biomarkers, including inflammatory factors, cytokines, and chemokines, may influence disease outcome^59^. Studies have found GZMB in early-stage lesions of CL patients, suggesting a role in disease pathogenesis. FARIA et al.^60^ reported higher percentages of GZMA^+^ cells and CD8^+^ GZMA^+^ cells in late-stage CL compared to early-stage, indicating that extensive tissue damage in the late-stage correlates with increased GZMA-expressing cells. In *L. braziliensis* infection, determining immune and inflammatory biomarkers is crucial for diagnosing early and late disease stages. Inflammatory factors like CXCL9 and CXCL10 show elevated expression in the delayed phase^61^. Mucocutaneous leishmaniasis is a severe type of *L. braziliensis* that destroys face structures. Notably, this severe appearance is partly caused by delayed disease diagnosis^62^. In this study, the ROC curve analysis of the GSE55664 dataset (early vs. late phase) identified two significant hub genes, GBP1 and CXCL10, highlighting their potential as biomarkers for early leishmaniasis diagnosis, consistent with previous studies (Supplementary Fig. 3; Fig. 9). However, experimental validation is necessary to confirm these findings and identify additional key biomarkers. Further studies and differential diagnostic tests for leishmaniasis are crucial.

One of the hub genes discovered in this study, which differs from those involved in inflammatory pathways, is the UBD gene. Although ubiquitination has not yet been deeply investigated in Leishmania, reports on particular cellular processes, genome-wide RNA interference screens, and other data indicate that these modifications are widely and critically important in these parasites^63,64^. Leishmania parasites utilize post-translational modifications, such as ubiquitination, to differentiate between their promastigote and amastigote forms. The Leishmania mexicana genome includes 2 E1 ubiquitin-activating genes, 13 E2 ubiquitin-conjugating genes, 79 E3 ubiquitin ligase genes, and 20 deubiquitinating cysteine peptidase genes; however, the functions of the E1, E2, and E3 enzymes remain unclear^65^. Previous research has investigated the role of DUBs and the parasite proteasome at different stages of the Leishmania life cycle^66,67^. Despite the importance of ubiquitination and deubiquitination enzymes in Leishmania differentiation, as well as the possibility of targeting these enzymes in developing new anti-leishmanial therapeutics, the Leishmania ubiquitination system is little understood. The UBD could serve as a potential target for anti-leishmania drug design, as the ubiquitin-proteasome system is a promising drug target in trypanosomatids and Leishmania species. Evidence suggests that selectively targeting this system in these parasites is feasible^64^. Since human and Leishmania enzymes differ, selective drugs may be identified, as human E1 inhibitors like TAK-243 are ineffective against trypanosomes^68^. Efforts to treat leishmaniasis have focused on both the effector proteasome and ubiquitination components^69^.

The miRNet database predicts potential miRNAs that can help researchers understand the biological activities of hub genes in leishmaniasis. This study identified five miRNAs associated with these hub genes: has-mir-146a-5p, has-mir-99b-5p, has-mir-129-2-3p, has-mir-27a-5p, and has-mir-21-3p. In *L. major*-infected human macrophages, miR-146a up-regulates and targets SMAD4 in the TGF-β signaling pathway to enhance parasite elimination^70^. System Studies identified miR-146a as a target in a model of Leishmania major infection. Nimsarkar et al.^70^ showed that some miRNAs, including miR-146a-5p and miR-146a-3p, were identified as key miRNAs affected by Leishmania infection in the inflammatory response. Ganguly et al.^71^ found that Leishmania can survive by transferring miR-146a from infected cells to resident cells, suppressing inflammation.

Bioinformatics studies face several limitations, including reliance on the quality of input data, incomplete or inaccurate biological databases, the complexity of biological systems, challenges in integrating diverse data types, and the need for experimental validation of computational predictions. These issues can impact the effectiveness and reliability of bioinformatics research, highlighting the importance of addressing these limitations to enhance the robustness and utility of computational approaches in advancing biological understanding^72–74^. However, it is necessary that the results obtained from our work are studied at the level of in vitro, in vivo or in the clinical phase to confirm the present research.

## Conclusion

Finally, three datasets of *L. braziliensis* were merged for bioinformatics studies, revealing three functional gene modules and seven hub genes. To understand the biological significance of these Hub genes in leishmaniasis, the miRNet database suggested possible miRNAs for these genes. We also investigated a drug interaction with hub genes using different databases. The results showed that most drugs have an inhibitory effect. These findings will aid in further exploring the mechanisms behind the occurrence and progression of leishmaniasis, as well as the identification of prospective targets for future diagnosis and treatment of leishmaniasis patients.

## Materials and methods

### Search strategy for the leishmaniasis microarray datasets

Datasets from high-throughput microarray and next-generation sequence functional genomics are submitted to GEO (Gene Expression Omnibus) by researchers around the world^75^. In this study, 58 datasets were searched in PubMed and GEO Database, the datasets were screened in terms of small sample size and reached 24 datasets, and then 15 datasets were selected by screening the datasets in terms of titles and abstracts, then 5 datasets were selected according to the DEGs, and finally 3 desired datasets were selected. The following keywords were used for searching: (“leishmaniasis“[MeSH Terms] AND “Homo sapiens” AND “Expression profiling by array“[Filter]). In order to be included, the datasets had to meet the following criteria: (1) they included both patients and normal controls, (2) the sample source was “skin tissue,” and (3) the differentially expressed genes (DEGs) with | logFC| > 1.5 and adjusted *P* < 0.05 were found from each dataset.

### Identification of DEGs in leishmaniasis

In the first step, we obtained the gene expression profiles for all the datasets included in the final analysis by downloading them from the GEO database. When many probes target the same gene, we compute the average scaled expression value for each gene. Next, the matrix file was extracted using the R GEOquery package, and quantile normalization was performed using the normalizeBetweenArrays function from the Limma package in the R software. Furthermore, we applied the criteria of |logFC| > 1.5 and adjusted *P* < 0.05 to identify the Differentially Expressed Genes (DEGs) in each dataset. This approach was done to compare the results with the RRA analysis.

### RRA integrated analysis

In RRA, by comparing the gene ranking in a random-ordered list with the baseline case, a higher gene rank will result in a lower P-value. It is important to note that RRA is only one way to analyze meta-analysis of multiple studies^76^. For the RRA analysis, the differentially expressed genes (DEGs) from each dataset were sorted and ranked based on their logFC values using the Limma package in the R software. These DEGs, which included both up-regulated and down-regulated genes, were then scored based on the ranked list and aggregated using R’s RRA package. In this approach, the adjusted P-value represents a probability that highly ranked genes in the datasets will be identified as DEGs. DEGs were identified using the criteria of |logFC| > 1.5 and adjusted *P* < 0.05.

### Functional annotation

Gene Ontology (GO) is a collaborative resource in bioinformatics that helps annotate gene sets and is categorized into three parts: biological process (BP), cellular component (CC), and molecular function (MF). Additionally, the Kyoto Encyclopedia of Genes and Genomes (KEGG)^19,20^ and WikiPath are databases that cover a wide range of biological signaling pathways. GO enrichment analysis, along with WikiPath and KEGG pathway analysis, play crucial roles in bioinformatics analysis by providing important insights. In this study, the Cluster Profile package in R software was utilized to perform enrichment analysis for the differentially expressed genes (DEGs). The criteria for DEG selection were adjusted *P* < 0.05 ^76,77^.

### Protein-protein Interaction (PPI) Network

To construct the PPI network for the identified differentially expressed genes (DEGs), the String database2 was utilized with a confidence parameter set to > 0.4. The PPI network was visualized using Cytoscape (v3.7.2), and the functional modules were identified with the molecular complex detection (MCODE) plugin in Cytoscape. Essential genes were determined using the CytoHubba plugin and ranked based on their degree scores. The hub genes were defined as the overlap between the genes in the PPI network with a degree score > 15, and by the RRA analysis, the top 15 DEGs (either down-regulated or up-regulated) were identified.

### Prediction of hub genes -miRNA interaction

The interaction of miRNAs and selected Hub genes was predicted using miRNet (https://www.mirnet.ca/), a visual network that depicts connections between miRNAs and target genes.

### Drug-hub genes interaction

The Drug-Gene Interaction Database (DGIdb; https://www.dgidb.org) and Drug Bank (https://go.drugbank.com) were used to choose drugs according to the hub genes that were offered as promising targets. The drugs that had received FDA (Food and Medicine Administration) approval were included in the final drug list. However, in the CTD database (https://ctdbase.org), we found a common drug for 6 gene hubs, which is classified in the investigational group.

### Evaluation of hub genes in the early and late phases of the disease

Among the three datasets examined in this study, only one dataset (GSE55664) divided the disease into two early and late phases. We analyzed this dataset separately to determine the fold change of their expression.

In the GSE55664 dataset, we considered three sets of genes, i.e., up-regulated DEGs, down-regulated DEGs, and hub genes, and these genes were subjected to ROC analysis in two phases, early and late.

## Electronic supplementary material

Below is the link to the electronic supplementary material.

Supplementary Material 1

## Data Availability

Data availability statementThe data described in this paper are available from the Corresponding author upon reasonable request.
