# Supplementary material for: Determination of key hub genes in Leishmaniasis as potential factors in diagnosis and treatment based on a bioinformatics study
Source: Sci Rep. 2024 Sep 28;14:22537. doi: 10.1038/s41598-024-73779-w (PMC11438978; doi:10.1038/s41598-024-73779-w)
Supplement: Supplementary file 1 — Supplementary Material 1 [file 41598_2024_73779_MOESM1_ESM.docx]

**Determination of Key Hub Genes in Leishmaniasis as Potential Factors in** [**Diagnosis**](https://ieeexplore.ieee.org/abstract/document/9718075/) **and Treatment Based on a Bioinformatics Study**

Mohsen Safaei^1^, Arash Goodarzi^1^, Zahra Abpeikar^1*^, Ahmad Reza Farmani^1^, Seyed Amin Kouhpayeh^2^, Sohrab Najafipour^3^, and Mohammad Hassan Jafari Najaf Abadi^4*^

1. Department of Tissue Engineering, School of Advanced Technologies in Medicine, Fasa University of Medical Sciences, Fasa, Iran

2. Department of Pharmacology, School of Medicine, Fasa University of Medical Sciences, Fasa, Iran

3. Department of Microbiology, Faculty of Medicine, Fasa University of Medical Sciences, Fasa, Iran

4. Department of Medical Biotechnology, School of Medicine, Shahid Sadoughi University of Medical Sciences and Health Services, Yazd, Iran

**Correspondence authors:**

Dr. Mohammad Hassan Jafari Najaf Abadi, PhD

Department of Medical Biotechnology, School of Medicine, Shahid Sadoughi University of Medical Sciences and Health Services, Yazd, Iran

Email: mohammadhassanj@gmail.com

Dr. Zahra Abpeikar, PhD

Department of Tissue Engineering, School of Advanced Technologies in Medicine, Fasa University of Medical Sciences, Fasa, Iran

Email: [zahraabpaikar@yahoo.com](mailto:zahraabpaikar@yahoo.com)


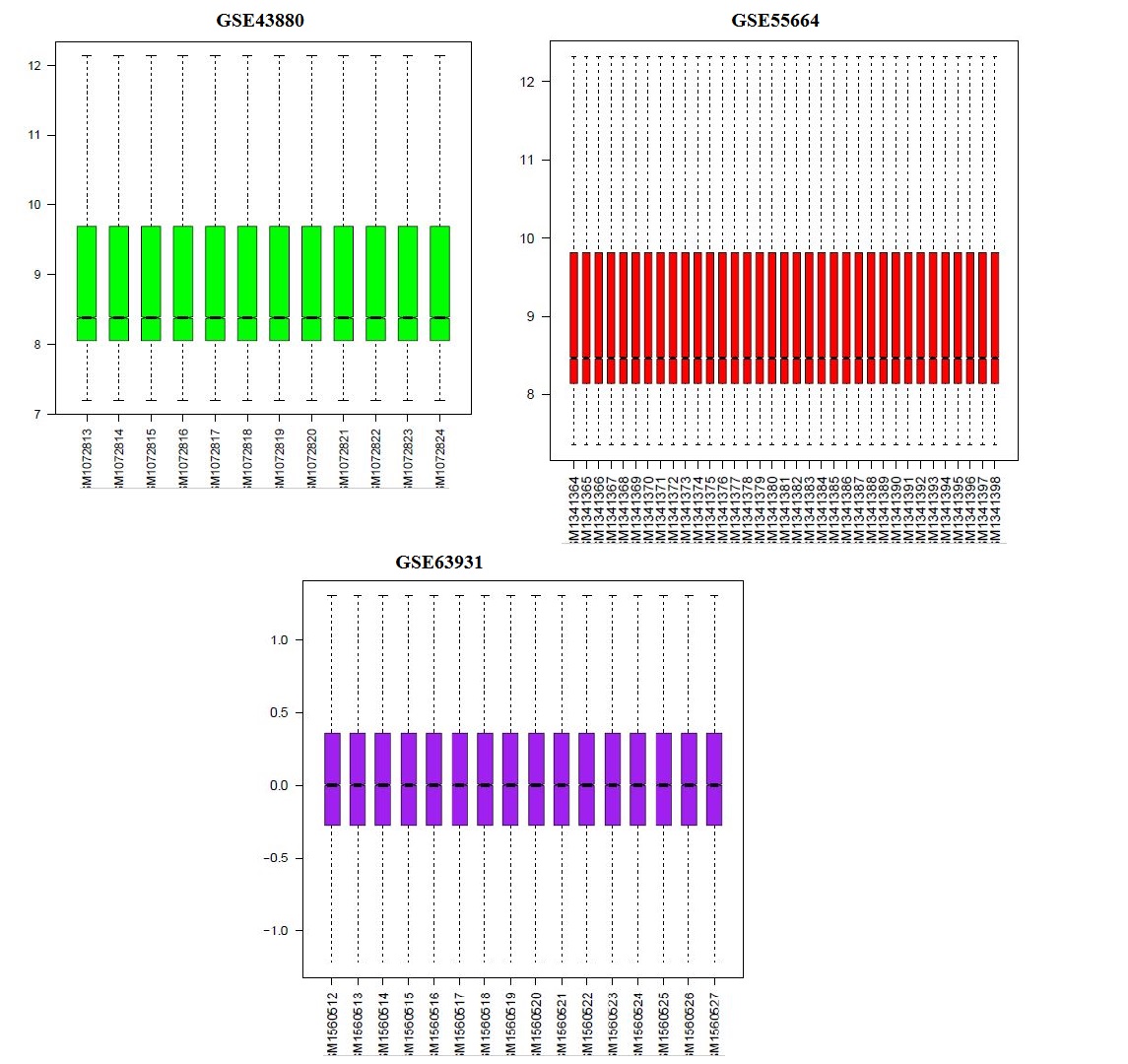


Supplementary Figure 1. Boxplot diagram for patient and normal samples for three datasets, where the absence of outlier data indicates the quality of the diagram.

Supplementary Table 1. The top 41 DEGs (upregulated or downregulated) found in the RRA analysis. The values in the heatmap indicate the logarithmic fold change in each dataset, as estimated by R software.

| **Gene name** | **GSE43880-DEG** | **GSE55664-DEG** | **GSE63931-DEG** |
| --- | --- | --- | --- |
| MMP1 | 5.765333 | 5.457243 | 12.46986 |
| MMP3 | 3.838605 | 4.694505 | 11.67869 |
| CXCL9 | 6.06756 | 6.418929 | 9.74137 |
| GBP5 | 6.233586 | 5.827936 | 9.268084 |
| CXCL10 | 6.036295 | 6.157283 | 9.173161 |
| CD38 | 4.088483 | 3.848627 | 8.996281 |
| IDO1 | 6.141872 | 5.967202 | 8.487511 |
| GZMB | 6.418302 | 5.88 | 7.796682 |
| CCL8 | 4.400034 | 5.020812 | 7.573137 |
| ADAMDEC1 | 4.564585 | 4.260959 | 7.460067 |
| GNLY | 4.590037 | 3.92451 | 6.948282 |
| NKG7 | 5.661488 | 4.988311 | 6.656218 |
| UBD | 4.843499 | 4.652014 | 6.542577 |
| GZMA | 5.830856 | 5.301054 | 6.258855 |
| GBP1 | 4.599462 | 4.391139 | 5.984713 |
| PRF1 | 4.885466 | 4.032781 | 5.961043 |
| IFNG | 3.981835 | 3.636692 | 5.936661 |
| GZMK | 5.287367 | 4.878622 | 5.916725 |
| CCL5 | 5.092704 | 4.617634 | 5.871397 |
| C1QB | 4.156372 | 4.508883 | 5.839074 |
| PIP | -6.56682 | -5.78342 | 1.142544 |
| SCGB2A2 | -5.22445 | -4.85551 | 0 |
| SCGB1D2 | -5.55444 | -5.14298 | 0 |
| DCD | -6.30668 | -5.34946 | 0 |
| ACSBG1 | -6.23724 | -3.23053 | -1.93549 |
| THRSP | -7.19912 | -4.0556 | -3.58588 |
| PON3 | -5.03405 | -2.80853 | -4.24374 |
| KRT15 | -3.5171 | -3.84909 | -4.40704 |
| PM20D1 | -6.39677 | -2.89244 | -4.4599 |
| C5orf46 | -4.55322 | -4.54219 | -4.63553 |
| HMGCS2 | -2.93673 | -2.81169 | -4.72557 |
| DAPL1 | -4.19716 | -4.1158 | -4.77311 |
| PSAPL1 | -4.32402 | -2.767 | -4.7769 |
| SERPINA12 | -3.57183 | -4.26325 | -5.30653 |
| GPT | -4.93578 | -2.2662 | -5.47559 |
| LCE5A | -2.65397 | -3.58455 | -5.49361 |
| AWAT2 | -6.31485 | -2.70581 | -6.09381 |
| KRT77 | -2.81171 | -3.5536 | -6.41878 |
| CHP2 | -2.81034 | -4.04476 | -6.66759 |
| FABP7 | -3.93901 | -3.17003 | -6.98262 |

Supplementary Table 2. Analysis of PPI networks. MCODE plugin in Cytoscape determined the top three modules (1,2 and 3) with the strongest scores.

| **Gene name** | **MCODE::Score (1)** | **MCODE::Node Status (1)** | **MCODE::Clusters (1)** |
| --- | --- | --- | --- |
| **Module 1** | | | |
| TRIM22 | 13 | Seed | Cluster 1 |
| IFI44 | 13 | Clustered | Cluster 1 |
| ISG15 | 13 | Clustered | Cluster 1 |
| IFIT2 | 13 | Clustered | Cluster 1 |
| IFI44L | 13 | Clustered | Cluster 1 |
| PARP14 | 13 | Clustered | Cluster 1 |
| SAMD9L | 13 | Clustered | Cluster 1 |
| MX1 | 13 | Clustered | Cluster 1 |
| IFI6 | 12 | Clustered | Cluster 1 |
| OAS2 | 11.89542 | Clustered | Cluster 1 |
| IFIT3 | 11.89542 | Clustered | Cluster 1 |
| RSAD2 | 11.89542 | Clustered | Cluster 1 |
| XAF1 | 11.89542 | Clustered | Cluster 1 |
| STAT1 | 11.89542 | Clustered | Cluster 1 |
| EPSTI1 | 11.89542 | Clustered | Cluster 1 |
| OASL | 11.86813 | Clustered | Cluster 1 |
| GBP1 | 11.2 | Clustered | Cluster 1 |
| GBP4 | 11 | Clustered | Cluster 1 |
| LAP3 | 10.71795 | Clustered | Cluster 1 |
| CXCL10 | 10.51648 | Clustered | Cluster 1 |
| **Module 2** | | | |
| KLRB1 | 10 | Seed | Cluster 2 |
| CD6 | 10 | Clustered | Cluster 2 |
| ICOS | 9.848485 | Clustered | Cluster 2 |
| CD96 | 9.848485 | Clustered | Cluster 2 |
| CD3E | 9.848485 | Clustered | Cluster 2 |
| IL2RB | 9.487179 | Clustered | Cluster 2 |
| CD3D | 9.230769 | Clustered | Cluster 2 |
| GZMB | 9.230769 | Clustered | Cluster 2 |
| ITK | 9.230769 | Clustered | Cluster 2 |
| SH2D1A | 9.230769 | Clustered | Cluster 2 |
| GNLY | 9.230769 | Clustered | Cluster 2 |
| CD3G | 9.230769 | Clustered | Cluster 2 |
| CXCR6 | 9 | Clustered | Cluster 2 |
| CTSW | 9 | Clustered | Cluster 2 |
| CXCL11 | 9 | Clustered | Cluster 2 |
| CXCL9 | 9 | Clustered | Cluster 2 |
| EOMES | 9 | Clustered | Cluster 2 |
| CXCR3 | 9 | Clustered | Cluster 2 |
| GPSM3 | 9 | Clustered | Cluster 2 |
| CCR1 | 9 | Clustered | Cluster 2 |
| CXCL13 | 9 | Clustered | Cluster 2 |
| CCR7 | 9 | Clustered | Cluster 2 |
| GZMH | 8.952381 | Clustered | Cluster 2 |
| NKG7 | 8.952381 | Clustered | Cluster 2 |
| PRF1 | 8.952381 | Clustered | Cluster 2 |
| CD2 | 8.163158 | Clustered | Cluster 2 |
| CD247 | 8.163158 | Clustered | Cluster 2 |
| GZMK | 8.163158 | Clustered | Cluster 2 |
| CCL8 | 8 | Clustered | Cluster 2 |
| FASLG | 8 | Clustered | Cluster 2 |
| CST7 | 8 | Clustered | Cluster 2 |
| SAMD3 | 8 | Clustered | Cluster 2 |
| **Module 3** | | | |
| KRTAP9-3 | 7 | Seed | Cluster 3 |
| KRTAP17-1 | 7 | Clustered | Cluster 3 |
| KRT35 | 7 | Clustered | Cluster 3 |
| KRT71 | 7 | Clustered | Cluster 3 |
| KRTAP9-8 | 7 | Clustered | Cluster 3 |
| KRT25 | 7 | Clustered | Cluster 3 |
| KRT27 | 7 | Clustered | Cluster 3 |
| KRTAP9-4 | 7 | Clustered | Cluster 3 |
| KRT32 | 6 | Clustered | Cluster 3 |
| KRT15 | 6 | Clustered | Cluster 3 |
| KRT19 | 6 | Clustered | Cluster 3 |
| KRT31 | 6 | Clustered | Cluster 3 |
| KRTAP19-1 | 6 | Clustered | Cluster 3 |

Supplementary Table 3. CytoHubba was employed to identify critical genes within the protein-protein interaction network, which were subsequently arranged based on their degree scores.

| **ClusteringCoefficient** | **Betweenness** | **Closeness** | **EPC** | **Degree** | **MNC** | **DMNC** | **MCC** | **Node_name** |
| --- | --- | --- | --- | --- | --- | --- | --- | --- |
| 0.28902 | 3324.848 | 120.4333 | 58.017 | 41 | 40 | 0.44797 | 1.63E+07 | CD2 |
| 0.27665 | 2549.307 | 119.2762 | 57.736 | 39 | 38 | 0.42279 | 1.59E+07 | CD247 |
| 0.21457 | 4092.81 | 119.0667 | 51.435 | 39 | 38 | 0.32792 | 15269 | TYROBP |
| 0.27778 | 5884.085 | 113.7929 | 42.007 | 37 | 27 | 0.65999 | 7.76E+09 | STAT1 |
| 0.36639 | 2388.326 | 116.4595 | 56.918 | 35 | 34 | 0.54318 | 1.37E+07 | GZMA |
| 0.34274 | 3712.749 | 113.2833 | 45.92 | 32 | 32 | 0.46956 | 5.19E+07 | CXCL10 |
| 0.27312 | 3441.3 | 116.7667 | 49.886 | 31 | 31 | 0.37024 | 490900 | CCR7 |
| 0.45517 | 405.6548 | 109.1929 | 55.372 | 30 | 30 | 0.61032 | 1.70E+07 | GZMK |
| 0.38424 | 1905.112 | 109.8167 | 52.815 | 29 | 28 | 0.5407 | 8244595 | CD3D |
| 0.36243 | 1553.935 | 113.9333 | 52.84 | 28 | 28 | 0.47485 | 8162324 | ITK |
| 0.46724 | 732.6087 | 107.1262 | 52.78 | 27 | 27 | 0.60468 | 1.01E+07 | CD8A |
| 0.39601 | 1225.25 | 111.9429 | 52.125 | 27 | 27 | 0.5125 | 9314922 | NKG7 |
| 0.37892 | 1933.497 | 110.7929 | 50.322 | 27 | 27 | 0.49038 | 8232072 | CD3G |
| 0.33048 | 1139.307 | 107.2429 | 47.539 | 27 | 27 | 0.4277 | 8686 | HCLS1 |
| 0.31624 | 1585.894 | 108.2929 | 48.371 | 27 | 26 | 0.43638 | 9393 | LAPTM5 |
| 0.33231 | 3102.681 | 116.15 | 47.069 | 26 | 26 | 0.42459 | 13146 | HCK |
| 0.31692 | 1296.096 | 108.4667 | 44.977 | 26 | 25 | 0.43285 | 8019 | SPI1 |
| 0.30769 | 1505.854 | 111.9333 | 45.972 | 26 | 26 | 0.39314 | 7602 | FCER1G |
| 0.28615 | 1886.288 | 112.9667 | 50.225 | 26 | 26 | 0.36562 | 12888 | CD48 |
| 0.34 | 1236.923 | 108.85 | 45.436 | 25 | 25 | 0.42865 | 414620 | CXCR6 |
| 0.49275 | 755.8415 | 102.8429 | 48.674 | 24 | 23 | 0.65857 | 9280947 | PRF1 |
| 0.42754 | 839.4543 | 107.5429 | 48.968 | 24 | 24 | 0.53152 | 7757510 | CD3E |
| 0.39855 | 1396.277 | 107.4333 | 46.949 | 24 | 24 | 0.49549 | 1134270 | CCL5 |
| 0.33696 | 868.7986 | 107.5929 | 46.07 | 24 | 24 | 0.41891 | 8370 | CD53 |
| 0.32246 | 942.3785 | 107.2262 | 46.096 | 24 | 23 | 0.43098 | 8811 | PLEK |
| 0.55336 | 780.1713 | 98.15952 | 35.973 | 23 | 23 | 0.67794 | 7.23E+09 | ISG15 |
| 0.52964 | 373.0302 | 102.5095 | 47.747 | 23 | 23 | 0.64888 | 9276026 | GZMH |
| 0.37154 | 519.9357 | 100.5595 | 42.983 | 23 | 23 | 0.45519 | 7346 | SASH3 |
| 0.35178 | 1721.166 | 107.1833 | 40.862 | 23 | 22 | 0.46481 | 12203 | MNDA |
| 0.34783 | 1831.599 | 109.8 | 42.359 | 23 | 23 | 0.42613 | 409544 | CCR1 |
| 0.29644 | 1204.831 | 107.0262 | 44.324 | 23 | 23 | 0.36318 | 908 | VAV1 |
| 0.68831 | 215.4131 | 94.84286 | 37.099 | 22 | 22 | 0.83038 | 7.76E+09 | XAF1 |
| 0.55844 | 878.7357 | 95.53333 | 36.367 | 22 | 21 | 0.72915 | 5.31E+08 | GBP1 |
| 0.38095 | 1103.458 | 108.6 | 48.473 | 22 | 22 | 0.45958 | 9224 | CD52 |
| 0.74286 | 184.6821 | 94.80952 | 35.58 | 21 | 21 | 0.88176 | 7.76E+09 | EPSTI1 |
| 0.72857 | 99.33743 | 91.57619 | 34.726 | 21 | 21 | 0.86481 | 7.76E+09 | IFIT3 |
| 0.68095 | 514.4568 | 95.10952 | 34.526 | 21 | 21 | 0.80828 | 7.72E+09 | IFI44L |
| 0.49048 | 764.1728 | 101.6429 | 45.679 | 21 | 20 | 0.63254 | 8084193 | SH2D1A |
| 0.30476 | 969.3395 | 103.6595 | 40.41 | 21 | 20 | 0.39303 | 3359 | MYO1F |
| 0.8 | 48.43454 | 91.07619 | 34.914 | 20 | 20 | 0.93345 | 7.76E+09 | OAS2 |
| 0.76842 | 276.2856 | 91.07619 | 34.184 | 20 | 20 | 0.89661 | 7.76E+09 | RSAD2 |
| 0.74211 | 277.4578 | 91.07619 | 35.824 | 20 | 20 | 0.8659 | 7.72E+09 | IFI44 |
| 0.72515 | 173.1626 | 90.80952 | 33.177 | 19 | 19 | 0.83088 | 7.22E+09 | SAMD9L |
| 0.60819 | 320.8209 | 93.15238 | 33.099 | 19 | 19 | 0.69687 | 4.10E+07 | GBP4 |
| 0.57895 | 179.8176 | 97.17619 | 44.402 | 19 | 18 | 0.72723 | 4840087 | IL2RB |
| 0.5731 | 695.2152 | 99.55952 | 42.17 | 19 | 17 | 0.79335 | 8790482 | GZMB |
| 0.52632 | 187.5124 | 99.49286 | 42.054 | 19 | 19 | 0.60306 | 3795444 | CD6 |
| 0.39181 | 1504.721 | 104.5333 | 37.71 | 19 | 19 | 0.44895 | 4934 | CYBB |
| 0.26901 | 3029.527 | 107.3667 | 36.872 | 19 | 13 | 0.57479 | 363614 | GPSM3 |
| 0.22222 | 2314.348 | 110.3595 | 39.798 | 19 | 17 | 0.30762 | 448 | LYN |
| 0.86928 | 28.60403 | 89.90952 | 32.774 | 18 | 18 | 0.97699 | 7.75E+09 | IFIT2 |
| 0.79085 | 47.65222 | 88.37619 | 33.395 | 18 | 18 | 0.88884 | 6.75E+09 | PARP14 |
| 0.64706 | 80.09184 | 101.8762 | 47.138 | 18 | 18 | 0.72723 | 4163760 | KLRB1 |
| 0.37255 | 1334.964 | 99.78333 | 33.24 | 18 | 17 | 0.46144 | 6039 | FCN1 |
| 0.36601 | 868.756 | 102.2667 | 32.186 | 18 | 18 | 0.41136 | 5864 | TLR8 |
| 0.26797 | 1362.078 | 105.5095 | 40.546 | 18 | 15 | 0.41061 | 5943 | IL10RA |
| 0.86029 | 31.48992 | 89.24286 | 32.81 | 17 | 17 | 0.94716 | 7.22E+09 | MX1 |
| 0.63235 | 329.2206 | 87.74405 | 30.671 | 17 | 17 | 0.6962 | 9.58E+08 | OASL |
| 0.5 | 953.7793 | 104.8667 | 36.471 | 17 | 17 | 0.55049 | 404184 | CXCL11 |
| 0.47059 | 1129.387 | 103.5333 | 35.215 | 17 | 16 | 0.57435 | 404065 | CXCL9 |
| 0.39706 | 1877.037 | 101.75 | 31.2 | 17 | 16 | 0.48461 | 3315 | GBP5 |
| 0.38971 | 793.876 | 100.3333 | 43.05 | 17 | 17 | 0.42905 | 2054 | PTPRCAP |
| 0.29412 | 841.5841 | 98.08333 | 32.969 | 17 | 16 | 0.35897 | 271 | ARHGAP9 |
| 0.775 | 43.21847 | 97.25952 | 41.479 | 16 | 16 | 0.8346 | 8789760 | GNLY |
| 0.68333 | 513.6887 | 90.29286 | 30.971 | 16 | 15 | 0.82122 | 1.52E+07 | LAP3 |
| 0.66667 | 452.575 | 99.65952 | 41.85 | 16 | 16 | 0.71794 | 1211042 | KLRD1 |
| 0.6 | 131.6246 | 99.01667 | 32.724 | 16 | 16 | 0.64614 | 409440 | CXCR3 |
| 0.48333 | 471.8587 | 96.73333 | 31.974 | 16 | 16 | 0.5205 | 7592 | CD14 |
| 0.425 | 459.3679 | 98.01667 | 36.356 | 16 | 16 | 0.45768 | 1142 | RASAL3 |


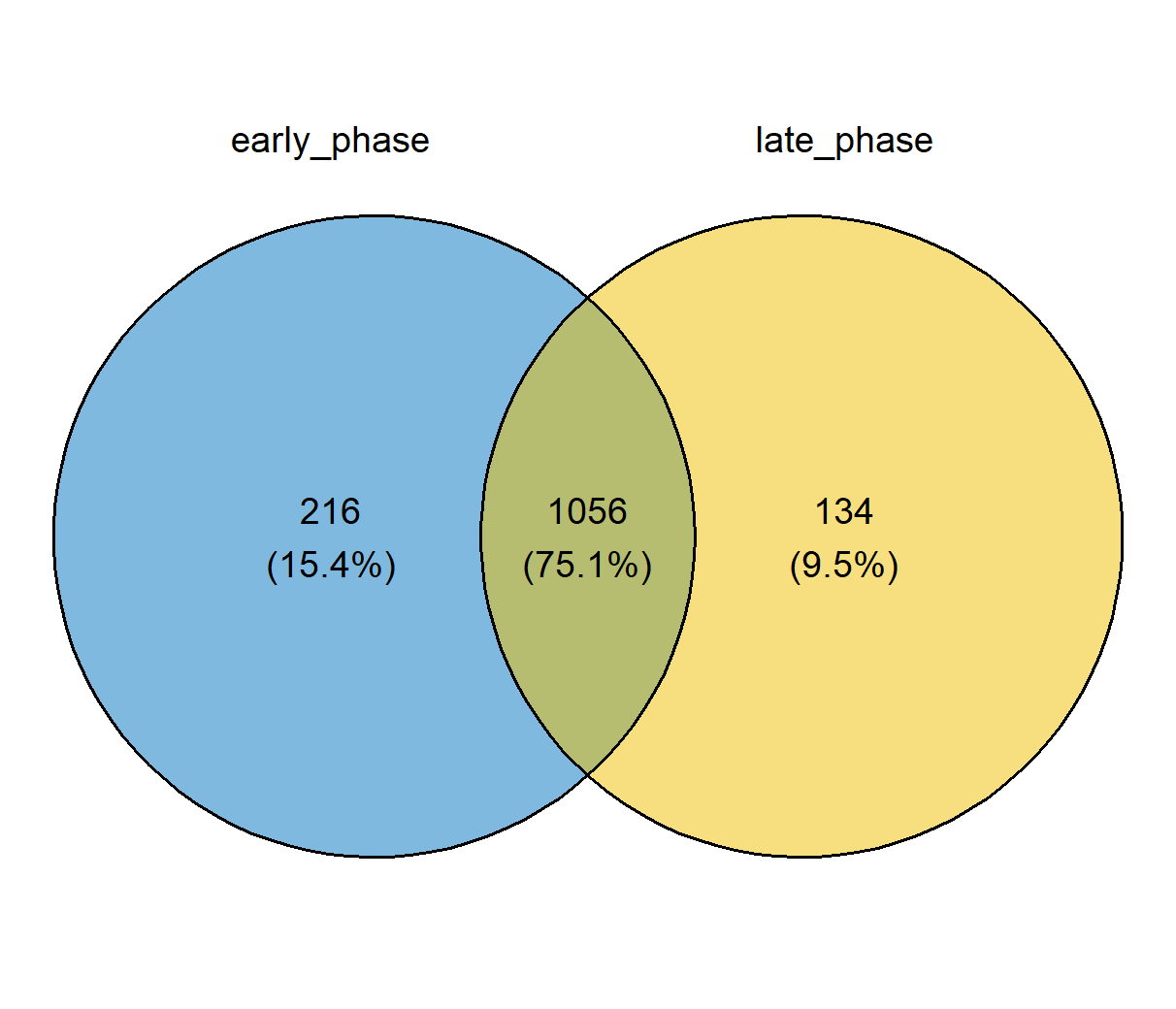


Supplementary Figure 2. The analysis results of the GSE55664 dataset showed that there are about 1056 common genes, including key hub genes, in both early and late stages of the disease.

Supplementary Table 4. Key hub genes are among the common genes of both the early and late phases of the disease, according to the results of the GSE55664 dataset analysis. Although their fold change rate is higher in the early phase and they showed some decrease in expression in the delayed phase.

| Gene | Early phase vs normal | Late phase vs normal | Fold change difference | Direction from early to late |
| --- | --- | --- | --- | --- |
| CXCL10 | 6.406628 | 6.040305 | 0.775757 | Down |
| GBP1 | 4.70411 | 4.25239 | 0.731171 | Down |
| GNLY | 4.172811 | 3.805432 | 0.775189 | Down |
| GZMA | 5.476534 | 5.222681 | 0.838654 | Down |
| GZMB | 6.275014 | 5.695642 | 0.669255 | Down |
| NKG7 | 5.142418 | 4.925341 | 0.860307 | Down |
| UBD | 4.888121 | 4.560261 | 0.796717 | Down |


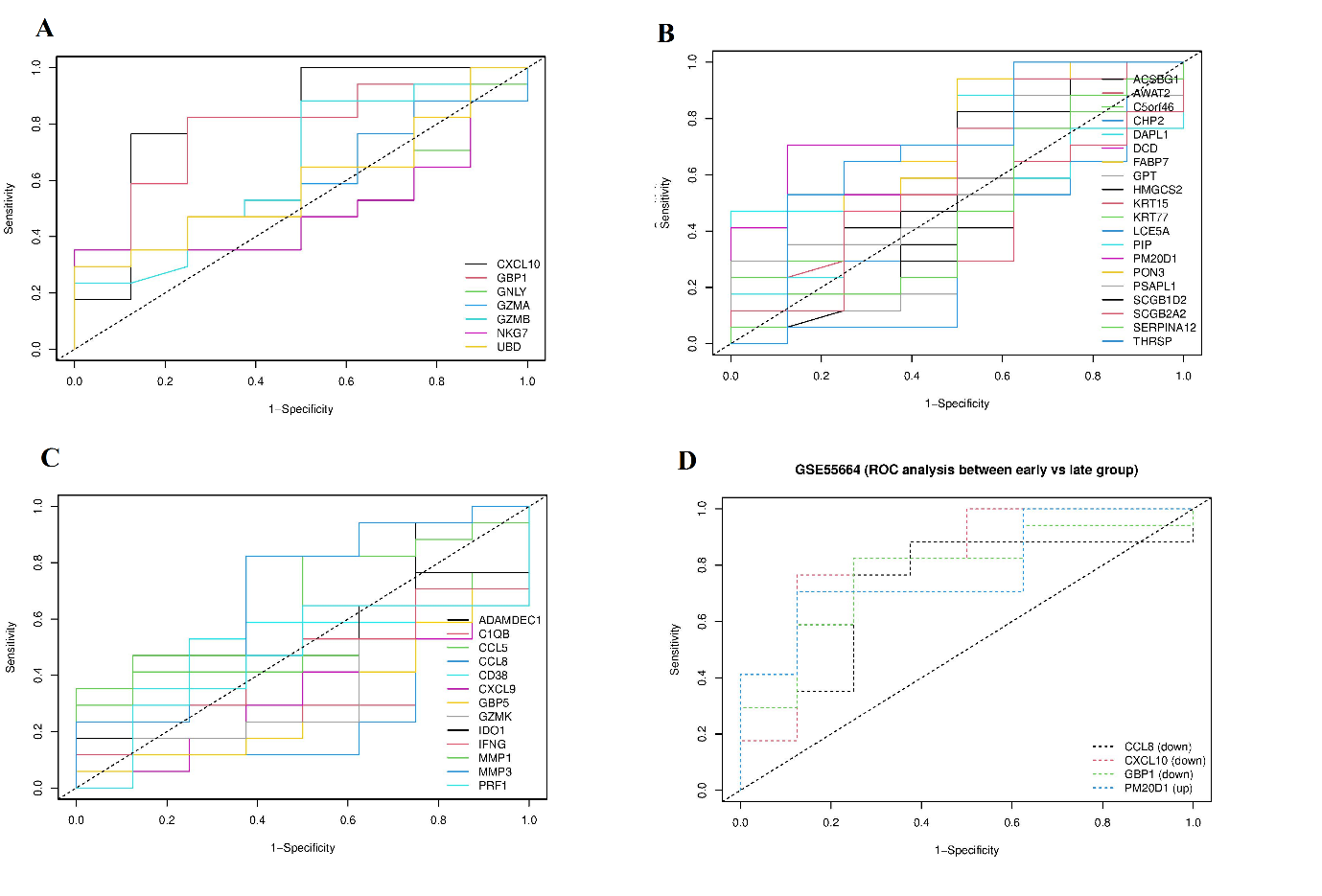


Supplementary Figure 3. ROC curve analysis of selected genes obtained from sharing three sets of genes (hub genes (A) down regulated DEGs (B), up regulated DEGs (C)) in GSE55664 dataset. 2 genes out of 4 obtained genes are members of hub genes and are significant at the P < 0.05 (CXCL10 and GBP1) (D).
